# Knowledge and Awareness of Hepatitis B Virus Infection in Nigeria

**DOI:** 10.5334/aogh.33

**Published:** 2019-04-11

**Authors:** Angela O. Eni, Mojisola G. Soluade, Oyewumi O. Oshamika, Oghenevwairhe P. Efekemo, Titilayo T. Igwe, Olabode A. Onile-ere

**Affiliations:** 1 Department of Biological Sciences, College of Science and Technology, Covenant University, Canaanland, Ota, Ogun State, NG; 2 West African Virus Epidemiology (WAVE) for Root and Tuber Crops, Covenant University Hub, Canaanland, Ota, Ogun State, NG

## Abstract

**Background:**

The World Health Organisation recently launched a campaign to reduce Hepatitis B Viral Infections by 80% globally. Achieving this goal is partly predicated on proper awareness of persons in regions of high transmission.

**Objective:**

The aim of this study was to assess the Hepatitis B Virus (HBV) infection knowledge status of persons across three states in Nigeria.

**Methods:**

A descriptive cross-sectional study among 758 persons selected by convenience sampling was conducted from March to July 2016. Structured questionnaires were administered to consenting participants and analysed using descriptive and inferential statistical methods in SPSS V20.

**Findings:**

Respondents showed average knowledge with a mean knowledge score of 4.85 ± 2.69 out of a max score of 9.00. Respondents belonging to the working class had significantly better knowledge (5.59 ± 2.34 p < 0.001) than respondents in other categories. High-risk behaviour such as having multiple partners was predominant among respondents belonging to a public institution. A total of 242 (31.96%) of study respondents were aware of the existence of a vaccine for HBV, whereas only 161 (21.2%) had received at least one dose of vaccination against HBV. Previous knowledge of HBV infection, previous HBV testing, and knowing someone who had HBV infection were predictors of HBV infection knowledge as well as vaccination.

**Conclusion:**

This study has shown the urgent need for intervention targeted at raising awareness about HBV infection and the existence of a vaccine.

## Introduction

Recent estimates show that Hepatitis accounts for approximately 1.3 million deaths annually, making it the seventh leading cause of death globally [1]. Hepatitis B and C are responsible for a large proportion of hepatitis mortality and morbidity, with over 90% of persons infected unaware of their condition, and as such they don’t seek treatment [2].

The World Health Organisation (WHO) estimates that about 60 million persons in the African region are currently infected with the Hepatitis B virus (HBV), accounting for 23% of the global Hepatitis B disease burden) [3]. The burden of Hepatitis B is on the increase even though effective vaccines to prevent the disease have existed since the 1980s [3]. This continued increase in burden is due to ineffective or nonexistence of hepatitis management programs in the sub-Saharan African region. The high mortality and morbidity that results is due in part to the fact that persons can live asymptomatically with the virus for up to 30 years; as such, testing is mostly conducted when disease becomes chronic and liver cirrhosis is already severe. Furthermore, there is a general lack of awareness with studies showing average to poor knowledge of hepatitis B virus infection and hepatitis vaccine among persons residing in regions of high risk [4,5,6]. To curtail this problem, in 2015 the WHO launched an ambitious goal to reduce HBV infections by 90% and increase global vaccine coverage to 90% [3]. Achieving this goal would require proper interventions targeted at behaviour change in regions of high prevalence.

This study sought to assess the knowledge of hepatitis B and associated factors among three different groups across three states in Nigeria.

## Methods

### Study Settings and Participants

We conducted a descriptive cross-sectional study from March to July 2016 across three major states in Nigeria: Lagos, Ogun and Abia States. The participants included were University Students from Covenant University, Bells University of Technology, Crawford University, Gateway Polytechnic Igbesa and the general public in Lagos and Abia State. Convenience sampling method was used.

### Study Instrument and Measures

The study instrument was a self-administered questionnaire designed after consulting previously published studies. The questionnaire consisted of the following two sections.

*Demographic Information.* Participants were asked to indicate their age group, marital status and the highest level of education attained.

*HBV Knowledge and Perception.* This section contained 29 questions assessing the knowledge and beliefs of participants toward HBV infection and vaccination. This section included nine questions used to access how much the respondent knew about HBV. One mark was given for each correct response, and zero marks were given for an incorrect or no response. Knowledge scores ranged from one to nine and were normalized by taking the percentage of correct responses. Respondents were divided into three categories based on their normalized knowledge scores; those scoring greater than 30 but less than or equal to 60 were classified as having average knowledge, whereas those scoring above 60 were classified as having good knowledge. Study respondents were also asked to provide vaccination information.

### Data Analysis

For better granularity, we created three strata based on the location and characteristics of the study respondent. Respondents from Covenant University, Bells University of Technology and Crawford University were grouped as Private Institution; respondents from Gateway Polytechnic Igbesa were grouped as Public Institution; and respondents who didn’t belong to a university, were in the working class and were from Abia and Lagos were grouped as Working Class.

Descriptive statistics were used to describe distributions. A chi-square test was used to assess the difference in proportion of independent variables with previous vaccination. Our continuous dependent variable was checked for normality using the Shapiro-Wilk and Kolmogorov-Smirnov tests alongside a histogram. To examine the relationships between our dependent (knowledge score) and independent variables, we performed either a Mann-Whitney test for independent variables with two categories or a Kruskal-Wallis test for independent variables with more than two categories. Pair-wise tests were performed for post hoc Kruskal-Wallis comparisons. Multivariate analysis using a multilinear regression were used to assess whether significant (p < 0.05) variables from the bivariate analysis predicted knowledge. Dummies of categorical variables were created prior to including them in the model. Multicolinearity of independent variables was assessed using a spearman correlation in a correlation matrix and variance inflation factors (VIF). Variables with VIF exceeding 5.0 were removed. All analysis was performed using SPSS 20 for windows, and p < 0.05 was considered statistically significant for all tests.

## Results

### Summary of Study Participants

A total of 805 responses were obtained, and 758 were included in the analysis based on completeness of demographic information. Table 1 shows the characteristic of respondents included in this survey. Of the 758 respondents, 347 (45.8%) were from a private institution, 233 (30.7%) were from a public institution and 178 (23.5%) didn’t belong to either group. Most respondents had never been married (80.3%), possessed higher education (92.3%) and were within the age range of 16–39 (88.7%).

**Table 1:** Summary of Study Participants.

		Private Institution		Public Institution		Working Class		Total	

		N	%	N	%	N	%	N	%

Age Group	<16	2	0.6%	0	0.0%	0	0.0%	2	0.3
	16–36	291	83.9%	233	100.0%	148	83.1%	672	88.7
	37–47	35	10.1%	0	0.0%	20	11.2%	55	7.3
	48–58	10	2.9%	0	0.0%	7	3.9%	17	2.2
	59–69	0	0.0%	0	0.0%	3	1.7%	3	0.4
	≥70	9	2.6%	0	0.0%	0	0.0%	9	1.2

Marital Status	Single	261	75.2%	223	95.7%	125	70.2%	609	80.3
	Married	77	22.2%	8	3.4%	50	28.1%	135	17.8
	Separated	9	2.6%	2	0.9%	3	1.7%	14	1.8

Education	Tertiary	311	89.6%	226	97.0%	163	91.6%	700	92.3
	Secondary	30	8.6%	7	3.0%	14	7.9%	51	6.7
	Primary	2	0.6%	0	0.0%	1	0.6%	3	0.4
	None	4	1.2%	0	0.0%	0	0.0%	4	0.5

Total		347	45.8%	233	30.7%	178	23.5%	758	100

### Knowledge of HBV

**Figure 1:**
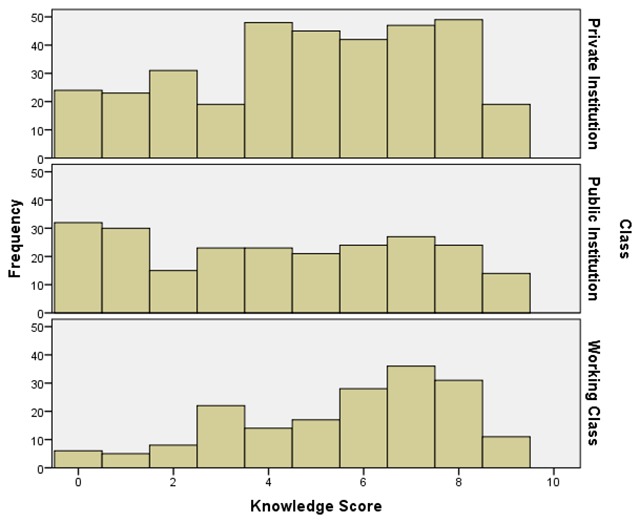
Distribution of Knowledge Scores.

The respondents in this study had average knowledge about HBV, with a mean knowledge score of 4.85 ± 2.69 (95% CI 4.66–5.04). See Figure 1. Knowledge scores across the three major groups differed significantly, with the working class (5.59 ± 2.340) having significantly (H = 24.617, p < 0.001) higher scores than respondents in private (4.91 ± 2.6) and public (4.19 ± 2.9) institutions. Post hoc tests showed that respondents from the Gateway Polytechnic Igbesa had significantly (p < 0.001) poorer knowledge than respondents from Covenant University, Abia State and Lagos State.

There was no significant difference observed for knowledge across the age groups (H = 6.823, p = 0.234) and levels of education (H = 2.986, p = 0.394). Persons who had heard about HBV (p < 0.001), had been previously tested (p < 0.001) or knew someone who had HBV (p < 0.001) were more likely to have better knowledge than those who did not. In addition, respondents who had been previously vaccinated (p = 0.01) or were aware of the HBV vaccine (p < 0.001) also had significantly better knowledge than those who had not received vaccination or heard about the vaccine (Table 2).

**Table 2:** Predictors of Knowledge in Respondents.

		Mean	SD	p-value

Age Group	<16	4.00	4.24	0.234
	16–39	4.79	2.67	
	37–47	5.51	2.87	
	48–58	5.12	2.80	
	59–69	7.00	2.00	
	≥70	4.33	2.45	

Class	Private School	4.91	2.60	<0.001
	Public School	4.19	2.90	
	General Public	5.59	2.34	

School	Bells	4.71	2.44	<0.001
	Covenant University	5.30	2.64	
	CRU	4.14	2.78	
	G. Poly	4.19	2.90	
	Lag	5.58	2.51	
	Umuahia	5.60	2.17	

Marital Status	Single	4.69	2.68	0.001
	Married	5.59	2.58	
	Separated	4.57	3.03	

Education	Tertiary	4.87	2.67	0.394
	Secondary	4.75	2.92	
	Primary	3.33	2.08	
	None	3.00	2.94	

Have you heard about HBV	No	2.68	2.51	<0.001
	Yes	5.72	2.22	

Have you been tested for HBV	No	4.49	2.69	<0.001
	Yes	6.58	1.85	

Do you know anyone currently infected	No	4.76	2.65	<0.001
	Yes	6.53	1.94	

Are you aware of the vaccine for prevention of HBV	No	4.23	2.67	<0.001
	Yes	6.49	1.81	

Have you been previously vaccinated against HBV	No	4.97	2.68	0.001
	Yes	5.83	2.37	

The highest proportion of correct responses was for questions regarding the aetiology of Hepatitis B (73.7%) and its ability to cause death (74%). See Table 3. Knowledge regarding the transmission routes was generally poor, with 56.5%, 35.4% and 31.4% knowing that bodily fluids, sexual intercourse and personal items, respectively, are routes of Hepatitis B transmission.

**Table 3:** Frequency of Correct Responses to Knowledge Assessment Questions.

	Private Institution		Public Institution		Working Class		Total	

	N	%	N	%	N	%	N	%

HBV is caused by …?	264	92.3%	153	87.4%	142	87.7%	559	73.7%
Do you know HB can affect liver and causes a chronic disease?	205	62.7%	114	55.6%	131	74.9%	450	59.4%
Can HBV cause yellowing of skin and eyes?	196	71.5%	105	61.0%	129	81.6%	430	56.7%
Do know HBV can lead to death?	268	82.2%	137	66.8%	156	89.7%	561	74.0%
Are you aware HBV is transmitted through blood and other bodily fluids?	211	65.5%	116	55.8%	101	65.6%	428	56.5%
Is HBV a STI?	122	37.8%	72	33.0%	74	43.8%	268	35.4%
Can HBV be transmitted from mother to child?	175	55.0%	110	52.6%	111	68.1%	396	52.2%
Are you aware that HB can be transmitted by sharing personal items?	111	33.5%	79	37.1%	48	28.4%	238	31.4%
Is Hepatitis B Curable?	107	38.6%	63	38.2%	68	46.3%	238	31.4%

### Vaccine Coverage

Only 242 (31.9%) of the respondents were aware of the presence of a vaccine against hepatitis B virus, and 161 (21.2%) had been previously vaccinated (Table 4). Persons who had good knowledge of HB (p = 0.003), had been previously tested (p < 0.001) and/or knew someone who was infected (p < 0.001) were more likely to have received a vaccine against Hepatitis B. As such, 182 (24%) and 181 (23.9%) of the study participants had more than one sexual partner and engaged in unprotected sex, respectively. Persons from a public institution had the highest proportion of respondents who engaged in unprotected sex (31.6%) or had more than one sexual partner (28.1%). Having more than one sexual partner (p = 0.663) and engaging in unprotected sex (p = 0.305) were not significantly associated with HBV vaccination.

**Table 4:** Previous Vaccination and Awareness.

		Private Institution		Public Institution		Working Class	

		N	%	N	%	N	%

Are you aware of the vaccine for prevention of HBV?	No	196	60.9%	159	77.9%	98	58.0%
	Yes	126	39.1%	45	22.1%	71	42.0%
Have you been previously vaccinated against HBV?	No	158	66.4%	127	79.4%	88	64.7%
	Yes	80	33.6%	33	20.6%	48	35.3%

### Multivariate Analysis

School of study was correlated with type of institution (VIF = 9.866) and as such was removed from the analysis. All other variables passed the test for multicollinearity at the VIF threshold of 5.

The independent predictors explained 35.2% of the variation in knowledge scores of study participants. The analysis showed that of the independent predictors, having previously heard about HBV was the most important contributor to knowledge, with persons in the category scoring 2.4 knowledge points (p < 0.0001) more than those who had not previously heard about HBV. Previous knowledge of the HBV vaccine was equally important, accounting for up to 1.171 knowledge points (p < 0.0001). Not being a part of a school, previous testing and knowing someone who was infected accounted for a cumulative 1.6 knowledge points in persons belonging to the three categories as compared to those who didn’t belong (Table 5).

**Table 5:** Factors Associated with Participant’s Knowledge.

	B	SE B	Beta	t	p-value	95% CI	VIF

(Constant)	3.184	0.317		10.053	<0.001	2.561 to 3.806	
Public institution	–0.046	0.272	–0.008	–0.167	0.867	–0.581 to 0.490	1.754
Private Institution	–0.202	0.237	–0.039	–0.855	0.393	–0.667 to .262	1.595
Single	–0.222	0.238	–0.036	–0.929	0.353	–0.690 to 0.247	1.141
Heard about HB	2.433	0.237	0.412	10.273	<0.001	1.968 to 2.898	1.220
Had been vaccinated	–0.337	0.227	–0.060	–1.485	0.138	–0.782 to .109	1.239
Tested for HB	0.553	0.260	0.095	2.129	0.034	0.043 to 1.063	1.526
Knowing someone who was infected	0.625	0.286	0.085	2.186	0.029	0.063 to 1.187	1.156
Aware of the HBV Vaccine	1.171	0.224	0.223	5.223	<0.001	0.730 to 1.611	1.381

## Discussion

In this study, we show average knowledge as well as poor vaccination coverage among study participants. To the best of our knowledge, there is a paucity of information on HBV knowledge among members of the general population in the study region. Most studies focus on populations for which Hepatitis B is an occupational hazard.

Awareness of HB was high (70%) in this study. This level of awareness, however, did not correlate with knowledge because only 46.4% of the study population had good knowledge of Hepatitis B virus (HBV) infection based on the knowledge assessment. Good knowledge in this study is lower than those in other studies, where they reported above 70% of respondents with good knowledge [7,8,9]. This asymmetry in awareness levels and knowledge could be indicative of low perceptions of HB risk among the study participants. In addition, our results showed that persons with high-risk behaviour, such as having unprotected sex with multiple sex partners, was not associated with HB knowledge or vaccine uptake. This again indicates a low perception of risk among such individuals.

Overall, our study participants had average knowledge with persons belonging to not belonging to a school having significantly better knowledge than students. The reason for this is not immediately clear based on the information collected, however analysis showed that members of the general public were also more likely to have been tested or vaccinated against HBV and as such could have received informal education about HBV infection. This relationship between vaccination and knowledge has been previously demonstrated [10,11]. Previous studies [5,12] have reported age as a predictor of knowledge, however in this study, age was not associated with knowledge. This is a result of the imbalance in our sampling because the age group 16–36 represented over two-thirds of the study population.

We found that persons who knew someone who had HBV infection were more likely to have been vaccinated and have higher scores. Again, this could be due to the increased perception of risk associated with knowing someone affected. This is in tandem with previous reports among Cambodian women living in the United States where the authors reported strong relationships between knowing someone currently infected, HB knowledge and HB testing [11]. In addition, a study conducted among health workers found higher vaccination rates among those with more knowledge of HBV infection [9].

Vaccination status as reported by the study respondents was lower than the recommended rate of 80% coverage by the WHO [3]. We found a high prevalence of high-risk behaviour such as having multiple sexual partners and having unprotected sex among study participants, however high-risk behaviours were not associated with vaccination status or being aware of the vaccine. This is a pointer to the need for urgent sensitisation of the persons in higher institutions of learning.

This study has a few limitations. We didn’t assess the influence of gender on knowledge and vaccination in our study population. We also did not directly measure the perceived risk of respondents, which may have a role to play in the knowledge of the different groups in this study [11].

## Conclusion

The results of this study have shown the urgent need for intervention in the areas of HBV awareness and vaccination. It is important to note that the general population consists of many subclusters with varying needs; as such, it would be expedient to tailor interventions to each cluster.

## References

[1] Abajobir AA, Abbafati C, et al. Global, regional, and national age-sex specific mortality for 264 causes of death, 1980–2016: A systematic analysis for the Global Burden of Disease Study 2016. Lancet. 2017; 390(10100): 1151–1210. DOI: 10.1016/S0140-6736(17)32152-9 28919116 PMC5605883

[2] Spearman CW, Afihene M, Ally R, et al. Hepatitis B in sub-Saharan Africa: Strategies to achieve the 2030 elimination targets. Lancet Gastroenterol Hepatol. 2017; 2(12): 900–909. DOI: 10.1016/S2468-1253(17)30295-9 29132759

[3] World Health Organization. Global Hepatitis Report, 2017. Geneva; 2017 http://apps.who.int/iris/bitstream/handle/10665/255016/9789241565455-eng.pdf;jsessionid=FF92E62958F285733BACE28B602BB99B?sequence=1 Accessed April10, 2018.

[4] Peteet B, Staton M, Miller-Roenigk B, Carle A and Oser C. Rural incarcerated women: HIV/HCV knowledge and correlates of risky behavior. Heal Educ Behav; 2018 DOI: 10.1177/1090198118763879 PMC11195302 29627991

[5] Adekanle O, Ndububa DA, Olowookere SA, Ijarotimi O and Ijadunola KT. Knowledge of hepatitis B virus infection, immunization with hepatitis B vaccine, risk perception, and challenges to control hepatitis among hospital workers in a Nigerian tertiary hospital. Hepat Res Treat. 2015: 1–6. DOI: 10.1155/2015/439867 PMC4320901 25685549

[6] Atiba BP, Ajao KO, Babalola RN, Awosusi AE, Ayeni OO and Ijadunola KT. Hepatitis B virus infection and its modes of prevention among clinical students of Obafemi Awolowo University (OAU), Ile-Ife, Nigeria. Afr J Med Med Sci. 2014; 43(Suppl): 31–37. http://www.ncbi.nlm.nih.gov/pubmed/26949778. Accessed April 11, 2018. 26949778

[7] Kesieme EB, Uwakwe K, Irekpita E, Dongo A, Bwala KJ and Alegbeleye BJ. Knowledge of hepatitis B vaccine among operating room personnel in Nigeria and their vaccination status. Hepat Res Treat. 2011: 1–5. DOI: 10.1155/2011/157089 PMC3199087 22028961

[8] Samuel SO, Aderibigbe SA, Salami TAT and Babatunde OA. Health workers’ knowledge, attitude and behaviour towards hepatitis B infection in Southern Nigeria. Int J Med Med Sci. 2009; 1(10): 418–424. http://www.academicjournals.org/ijmms. Accessed April 11, 2018.

[9] Hassan M, Awosan KJ, Nasir S, et al. Knowledge, risk perception and hepatitis B vaccination status of healthcare workers in Usmanu Danfodiyo University Teaching Hospital, Sokoto, Nigeria. 2016; 8(4): 53–59. DOI: 10.5897/JPHE2015.0795

[10] Cvjetkovic SJ, Jeremic VL and Tiosavljevic DV. Knowledge and attitudes toward vaccination: A survey of Serbian students. J Infect Public Health. 2017; 10(5): 649–656. DOI: 10.1016/J.JIPH.2017.05.008 28669785

[11] Betsch C and Wicker S. E-health use, vaccination knowledge and perception of own risk: Drivers of vaccination uptake in medical students. Vaccine. 2012; 30(6): 1143–1148. DOI: 10.1016/j.vaccine.2011.12.021 22192850

[12] Hajarizadeh B, Wallace J, Richmond J, Ngo N and Enright C. Hepatitis B knowledge and associated factors among people with chronic hepatitis B. Aust N Z J Public Health. 2015; 39(6): 563–568. DOI: 10.1111/1753-6405.12378 26095536

